# Metabolic Myopathies and HyperCKemia in Adulthood: A Clinical Approach to Diagnosis and Management

**DOI:** 10.3390/jcm15052070

**Published:** 2026-03-09

**Authors:** Loai A. Shakerdi

**Affiliations:** 1 School of Public Health, University College Dublin, D04 V1W8 Dublin, Ireland; loai.shakerdi1@ucd.ie; 2 National Centre for Inherited Metabolic Disorders, Mater Misericordiae University Hospital, Eccles St, Phibsborough, D07 R2WY Dublin, Ireland

**Keywords:** exercise intolerance, glycogen storage disease, creatine kinase, rhabdomyolysis, fatty acid oxidation disorders, mitochondrial disease

## Abstract

Background: HyperCKemia, defined as elevated serum creatine kinase, commonly reflects muscle injury but may also indicate underlying metabolic disease. Metabolic aetiologies, including glycogen storage disorders, fatty acid oxidation defects, mitochondrial cytopathies, and purine metabolism disorders, are clinically important owing to diagnostic complexity, therapeutic implications, and potential reversibility. Objective: To summarise current evidence on metabolic causes of hyperCKemia in adults, with emphasis on disorders of carbohydrate, lipid, and purine metabolism and mitochondrial disease. Methods: Semi-systematic narrative review of pathophysiology, clinical features, diagnostic approaches, and management of metabolic disorders associated with hyperCKemia. Results: Metabolic myopathies often present with nonspecific or exercise-related symptoms, with creatine kinase levels ranging from mild-to-severe elevations. Conditions such as McArdle disease, carnitine palmitoyltransferase II deficiency, and mitochondrial cytopathies demonstrate characteristic metabolic vulnerabilities leading to episodic or persistent hyperCKemia. Medications, including statins and antiretrovirals, may precipitate symptoms in predisposed individuals. Diagnosis requires a structured, multidisciplinary approach incorporating biochemical testing, genetic analysis, functional studies, and muscle biopsy. Many causes are amenable to targeted therapy, including dietary modification, endocrine correction, and medication withdrawal. Conclusion: Metabolic causes of hyperCKemia are under-recognised but clinically significant. Early identification allows targeted treatment and prevention of complications.

## 1. Introduction

HyperCKemia is defined by the Nordic Reference Interval Project as persistent creatine kinase (CK) concentrations ≥ 210 U/L in women, ≥400 U/L in men under 50 years, and ≥280 U/L in men aged 50 years and older [1]. CK is a key enzyme in cellular energy metabolism, and its elevation typically reflects muscle injury or dysfunction. The differential diagnosis is broad, encompassing physiological, pharmacological, inflammatory, endocrine, and inherited causes. Strenuous exercise is the most common non-traumatic trigger, while medications—particularly statins, fibrates, and certain antipsychotics—are frequent pharmacological contributors and may result in drug-induced myopathy. Endocrine disorders, especially hypothyroidism, as well as immune-mediated necrotizing myopathy, should also be considered in patients with persistent or unexplained CK elevation [2,3,4]. Importantly, a range of inherited metabolic diseases (IMDs) may present with hyperCKemia and are of particular clinical relevance given their potential reversibility and disorder-specific therapeutic implications.

The prevalence of IMDs among patients with persistent hyperCKemia is increasingly recognised. Many cases initially labelled as “idiopathic” are subsequently found to have an underlying genetic or metabolic basis [2]. In a cohort of 169 patients, next-generation sequencing (NGS) panels targeting muscle-specific genes yielded a definitive molecular diagnosis in approximately 36% of cases, with comparable detection rates across presentations including muscle weakness (37%), recurrent rhabdomyolysis (33%), and idiopathic hyperCKemia (31%) [5]. Similarly, a retrospective study of 1302 patients with acute CK levels > 2000 IU/L identified suspected genetic disease in nearly 15%, with pathogenic variants confirmed in 37% of this subgroup across 22 genes [6]. Clinical features suggestive of an IMD include marked CK elevations (>5–30× the upper limit of normal), significant fluctuation in CK levels, and recurrent rhabdomyolysis precipitated by exercise or fasting.

Despite the frequency of hyperCKemia as a laboratory abnormality, recognition of its metabolic causes remains challenging due to phenotypic heterogeneity and overlap with other neuromuscular disorders. This review therefore aims to synthesise current evidence on the metabolic aetiologies and management of hyperCKemia in adults.

### Literature Search and Evidence Appraisal

This review incorporates a semi-systematic narrative synthesis to summarise clinically relevant evidence regarding metabolic causes of hyperCKemia. A structured literature search was conducted in MEDLINE/PubMed, Embase, and the Cochrane Library from database inception to January 2026. Search terms combined controlled vocabulary and keywords related to hyperCKemia. Reference lists of relevant reviews, neuromuscular guidelines, and metabolic disease consensus statements were manually screened to identify additional pertinent publications. Eligible sources included clinical practice guidelines, consensus statements, randomised controlled trials, systematic reviews, cohort studies, and case–control studies. Adult studies were prioritised; paediatric data were included when adult evidence was limited but pathophysiology and management principles were transferable. Single case reports and very small case series were excluded unless they described rare but clinically important presentations or adverse effects. Publications lacking clinically relevant outcome data were excluded.

Evidence was prioritised according to methodological quality and clinical applicability. Given the rarity and heterogeneity of inherited metabolic myopathies, high-level comparative trials are limited; therefore, mechanistic evidence and consistent observational findings were incorporated where appropriate. Evidence certainty was interpreted in alignment with GRADE principles: high (randomised trials/robust longitudinal data), moderate (consistent cohort or mechanistic evidence), low (small studies or indirect outcomes), and very low (expert opinion or experimental therapies).

## 2. Physiology and Clinical Significance

**Figure 1:**
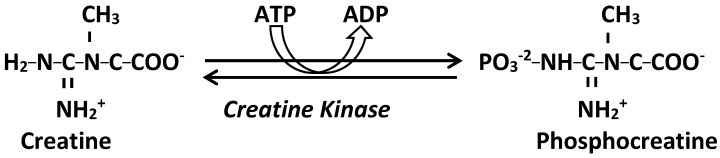
Biochemical reaction catalysed by creatine kinase (CK). Abbreviations: ATP, Adenosine Triphosphate; ADP, Adenosine Diphosphate. In the mitochondria, the reaction usually moves right, producing phosphocreatine (energy storage). In the cytosol (during exercise): The reaction moves, left producing ATP (energy usage).

CK is a cytosolic and mitochondrial enzyme that catalyses the reversible transfer of a phosphate group from adenosine triphosphate (ATP) to creatine, forming adenosine diphosphate (ADP) and phosphocreatine [7] (Figure 1). CK is a dimer molecule and occurs in three distinct isoenzyme forms electrophoretically: CK-MM (muscle), CK-MB (cardiac), and CK-BB (brain), with CK-MM comprising the majority of circulating CK in adults [8]. Macro-CK is a common macroenzyme with a higher molecular mass than normal serum CK. It occurs in two forms: macro-CK type 1, an enzyme–antibody complex (>200 kDa) usually composed of CK-BB bound to IgG (occasionally IgA or rarely IgM), and macro-CK type 2, a non–immunoglobulin-bound polymer of mitochondrial CK (>300 kDa). Both forms can falsely elevate CK-MB activity [9].

Acute CK elevation reflects metabolic stress, including intense or prolonged exercise, fasting, intercurrent illness, or myotoxic medications, and may present with myalgia, weakness, cramps, or rhabdomyolysis. In contrast, patients with lipid, glycogen, or mitochondrial myopathies may exhibit persistent hyperCKemia without overt symptoms. Recognition of acute versus chronic CK elevation patterns aids diagnosis, and distinction of metabolic myopathies from inflammatory or dystrophic muscle disease.

Serum CK typically rises within 2–12 h of muscle injury [10,11], peaks at 24–72 h [12], and normalises within 3–5 days, though recovery may take up to 7–10 days after severe rhabdomyolysis or sustained exertion [2]. A CK level ≥ 3–5 times the upper limit of normal is commonly used as a clinical threshold [13], although peak CK does not reliably predict severity or risk of acute kidney injury (AKI) [14].

Alanine aminotransferase (ALT) and aspartate aminotransferase (AST) are abundant in skeletal muscle and frequently rise alongside CK after muscle injury [15,16]. CK increases earliest and most prominently, while AST and ALT peak later (days 3–4 and 4–5, respectively). In rhabdomyolysis, AST typically exceeds ALT, producing an AST/ALT ratio > 1 [17]. Aminotransferases may remain elevated for up to three weeks after CK normalisation, potentially mimicking liver disease if muscle injury is unrecognised [18].

### 2.1. Metabolic Myopathies

Metabolic myopathies are a group of IMDs characterised by defects in enzymatic pathways involved in energy production within skeletal muscle. The pathogenesis of hyperCKemia in metabolic myopathies arises from impaired ATP generation, increased muscle cell fragility, and the subsequent leakage of CK from myocytes into the circulation [19]. While some metabolic myopathies are present in childhood, others manifest in adulthood, often with episodic or exercise-induced symptoms.

### 2.2. Glycogen Storage Diseases (GSDs)

#### 2.2.1. Glycogen Storage Disease Type II (GSD II; Pompe Disease)

GSD II is an autosomal recessive (AR) disorder arises from mutations in the *GAA* gene, leading to deficiency of lysosomal acid α-glucosidase [20]. This enzyme cleaves 1,4 and 1,6 linkages in glycogen. Deficiency of the enzyme results in glycogen accumulation. In adults, GSD II the disease manifests as progressive limb-girdle muscle weakness, respiratory insufficiency, and hyperCKemia. GSD II falls under two categories, GSDs and Lysosomal storage diseases (LSDs). In late-onset Pompe disease (LOPD), the residual enzyme activity is between 3 and 30% of normal. LOPD typically have increased CK values (between 1.5 and 15 times the upper limits of normal in adults) [21,22].

#### 2.2.2. Glycogen Storage Disease Type III (GSD III; Cori-Forbes Disease)

GSD III is an AR disorder caused by biallelic pathogenic variants in the *AGL* gene, resulting in deficiency of the glycogen debranching enzyme (GDE). GSD IIIA is the most common subtype, present in about 85% of affected individuals; it manifests with liver, cardiac, and muscle involvement. GSD IIIB mainly affects the liver [23,24].

#### 2.2.3. Glycogen Storage Disease Type V (GSD V; McArdle Disease)

GSD V is an AR disease; caused by mutations in the *PYGM* gene encoding the enzyme muscle glycogen phosphorylase myophosphorylase. Enzyme deficiency results in exercise intolerance, muscle cramps, and recurrent rhabdomyolysis. A hallmark of McArdle disease is the chronic elevation of serum CK levels. HyperCKemia is often present at rest and markedly increased following exertion. A 2020 study involving 60 patients demonstrated this consistency, with 100% of participants exhibiting CK levels 5 to 18 times higher than the upper reference limit [25].

#### 2.2.4. Glycogen Storage Disease Type VII (GSD VII, Tarui Disease)

GSD VII is an AR metabolic disorder caused by mutations in the *PFKM* gene. The defective enzyme, phosphofructo-1-kinase (PFK). Enzyme deficiency impairs glycolysis, resulting in exercise intolerance, myalgia, and occasional myoglobinuria. HyperCKemia is often observed, particularly after physical activity [26,27].

#### 2.2.5. Glycogen Storage Disease Type IX (GSD IX)

GSD IX is due to impaired activity of phosphorylase kinase (PhK), leads to clinical features overlapping with other GSD s. GSD IX subtypes IXa, IXb, and IXc arise from defects in the liver-specific α (*PHKA2*), β (*PHKB*), and γ (*PHKG2*) subunits of PhK. GSD IX is genetically heterogeneous: subtypes IXa and IXd are X-linked conditions resulting from variants in *PHKA2* and *PHKA1*. Subtypes IXb and IXc, caused by *PHKB* and *PHKG2* variants, are inherited in an AR manner. Clinical features include hepatomegaly, growth restriction, hypoglycaemia, muscle weakness, cramps, and persistent or exercise-induced hyperCKemia [28].

### 2.3. Fatty Acid Oxidation Disorders (FAOD)

Defects of mitochondrial fatty acid metabolism may result in severe bioenergetic imbalance. Patients may present with muscle pain, hypotonia, peripheral neuropathy, and cardiomyopathy [29,30,31]. Acute metabolic decompensation may develop in conditions associated with increased energy demand such as fasting, severe illness or infection.

#### 2.3.1. Carnitine Palmitoyltransferase II (CPT2) Deficiency

CPTII deficiency is an AR FAOD [32]. It is the most common inherited disorder of long-chain FAOD. CPTII deficiency has three clinical phenotypes. The adult myopathic form presents with recurrent episodes of muscle pain, weakness, and rhabdomyolysis, often triggered by prolonged exercise, fasting, or illness. Symptoms may develop at times of increased energy demand. HyperCKemia is typically episodic, coinciding with attacks, but may be elevated at baseline in some patients [33].

#### 2.3.2. Long-Chain 3-Hydroxyacyl-CoA Dehydrogenase Deficiency (LCHADD)

Long-Chain 3-hydroxyacyl-CoA dehydrogenase deficiency is a mitochondrial defect of β-oxidation of long chain fatty acids caused by mutations in the alpha subunit of the hydroxy acyl-CoA dehydrogenase (*HADHA*) gene. LCHAD deficiency is inherited in an AR manner. CK and transaminases can be used as a parameter for monitoring treatment [34,35,36,37,38].

#### 2.3.3. LPIN1 Deficiency

LPIN1 deficiency is an AR disease caused by biallelic mutations in *LPIN1* [39]. *LPIN1*, encodes the enzyme phosphatidic acid (PA) phosphohydrolase that catalyses the dephosphorylation of phosphatidic acid to yield diacylglycerol. Lipin-1 deficiency is a common cause of early-onset rhabdomyolysis [30].

Deficiencies in very-long-chain acyl-CoA dehydrogenase (VLCAD), and primary carnitine deficiency can also lead to exercise-induced or persistent hyperCKemia, though these are less common in adults [40,41].

#### 2.3.4. Mitochondrial Cytopathies

Mitochondrial disorders comprise a heterogeneous group of conditions arising from mutations in either mitochondrial DNA or nuclear genes encoding proteins essential for oxidative phosphorylation. HyperCKemia is encountered in mitochondrial myopathies, mitochondrial encephalomyopathy, lactic acidosis, and stroke-like episodes (MELAS), and progressive external ophthalmoplegia (PEO). Notably, CK elevations in MELAS and PEO are typically mild, often remaining within the reference range or rising to less than five times the upper limit of normal [42,43].

#### 2.3.5. Disorders of Purine Metabolism—Myoadenylate Deaminase Deficiency

Adenosine monophosphate deaminase comprises a family of isoforms that catalyse the conversion of adenosine monophosphate (AMP) to inosine monophosphate (IMP) and ammonia, marking the first step of the purine nucleotide cycle. This family includes three primary members with distinct tissue distributions: *AMPD1* is predominantly expressed in skeletal muscle, *AMPD2* is found across various non-muscle tissues, and *AMPD3* is localised mainly in erythrocytes [44]. Deficiency in AMPD1 is relatively common in the general population and may be asymptomatic or manifest as exercise intolerance, muscle pain, and mild hyperCKemia [45].

### 2.4. Diagnostic Approach to HyperCKemia in Adults (Figure 2)

The diagnostic evaluation of hyperCKemia should follow a structured and systematic approach to distinguish transient or benign elevations from inherited metabolic or neuromuscular disorders. Early recognition is important, as timely diagnosis enables targeted management, reduces the risk of recurrent rhabdomyolysis, and mitigates long-term complications.

**Figure 2:**
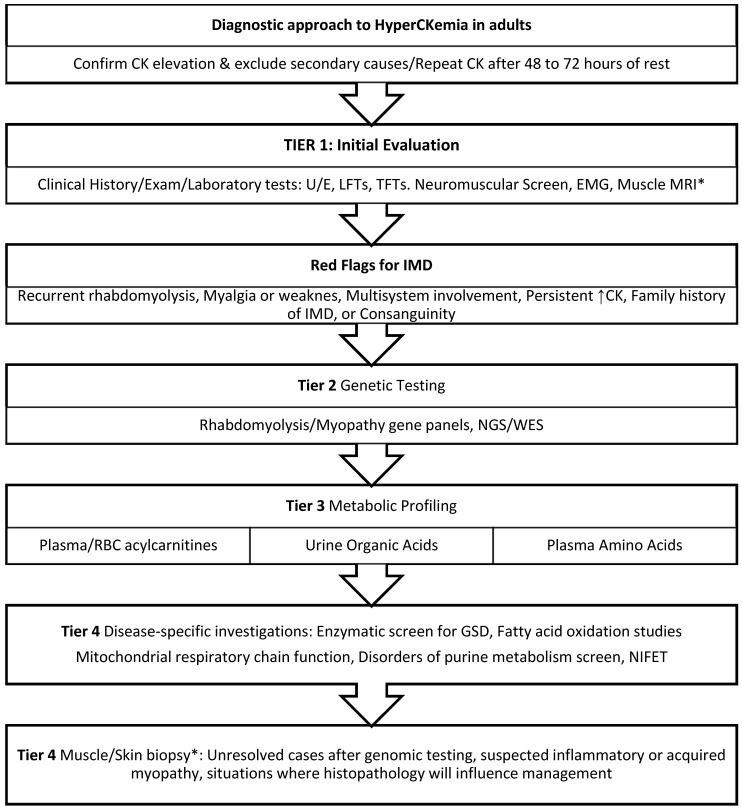
Diagnostic approach to HyperCKemia in adults. Abbreviations: CK, creatine kinase; EMG, electromyography; MRI, magnetic resonance imaging; NIFET, non-ischaemic forearm exercise test; NGS, next-generation sequencing; WES, whole-exome sequencing; U&E, urea and electrolytes; LFTs, liver function tests; TFTs, thyroid function tests; IMD, inherited metabolic disorder. * Muscle/Skin biopsy, EMG, and Muscle MRI might be performed at any stage of the diagnostic pathway.

Initial assessment should confirm persistence of CK elevation by repeat testing after 48–72 h of rest and exclude reversible causes, including recent strenuous exercise, medications, substances with potential myotoxic effects, endocrine disorders, electrolyte abnormalities, and intercurrent systemic illness.

Subsequent evaluation should be guided by the clinical context, including symptom profile (e.g., myalgia, cramps, exercise intolerance, or rhabdomyolysis), triggering factors, family history, and associated systemic features such as cardiac, hepatic, or neurological involvement. Persistent CK elevation or a suggestive phenotype should prompt consideration of an IMD or genetic myopathy.

Baseline investigations may include renal and hepatic function, thyroid function, electrolytes, fasting glucose, and urinalysis for myoglobin when clinically indicated. Electromyography (EMG) serves as an adjunctive tool rather than a mandatory first-line test; it can distinguish myopathic from neurogenic processes, identify inflammatory or necrotising myopathies, and guide muscle biopsy site selection when histopathology is required.

Recent literature supports an evolving genetics-first approach in adults with suspected inherited myopathies, reflecting the high diagnostic yield and non-invasive nature of next-generation sequencing (NGS), including targeted gene panels, whole-exome sequencing (WES), and whole-genome sequencing (WGS) [46]. Muscle biopsy should be reserved for cases in which genetic testing is non-diagnostic, an acquired myopathy is suspected, or histological clarification will influence management. In asymptomatic or minimally symptomatic hyperCKemia, biopsy abnormalities are common but often nonspecific; histopathological changes were reported in 55% of cases in one cohort, whereas a definitive diagnosis of muscular dystrophy was established in only 8% [47].

In GSDs, enzyme assays performed on leukocytes, dried blood spots, or muscle biopsy specimens remain the definitive method for quantifying residual activity of enzymes such as myophosphorylase or acid α-glucosidase [48]. Metabolic screening with plasma acylcarnitine profiling, urine organic acid analysis, and plasma amino acids can provide biochemical evidence of mitochondrial dysfunction or FAOD [49,50,51,52]. Functional studies, including in vitro probe assays and fatty acid oxidation flux studies in cultured fibroblasts or lymphocytes, provide a critical physiological bridge when genetic variants of uncertain significance are identified or when clinical suspicion remains high despite inconclusive genomic findings. In mitochondrial disease, evaluation of the respiratory chain through spectrophotometric enzyme assays or oxygen consumption rate measurements using extracellular flux analysis enables characterisation of specific complex deficiencies [53]. Non-ischaemic forearm exercise test (NIFET) remains a useful screening tool in patients with exercise intolerance and suspected metabolic myopathies [54].

### 2.5. Management of Acute HyperCKemia and Rhabdomyolysis

In acute CK elevation, management focuses on aggressive hydration, prompt treatment of precipitating factors, and rapid correction of energy deficiency.

In GSDs, rhabdomyolysis management prioritises rapid extracellular volume expansion with isotonic saline (0.9% NaCl) to maintain adequate urine output, alongside intravenous (IV) dextrose to provide an immediate glucose source and suppress ongoing muscle breakdown.

In fatty acid oxidation disorders, prompt administration of IV dextrose (typically 10%) with electrolyte supplementation is a cornerstone of acute management during catabolic stress. Beyond providing an immediate energy substrate, glucose administration has been shown to improve metabolic stability and is thought to confer benefit through insulin-mediated suppression of lipolysis, thereby reducing mobilisation of long-chain fatty acids and limiting accumulation of toxic long-chain acylcarnitines implicated in metabolic instability and muscle injury [55,56,57,58,59]. When tolerated, oral medium-chain triglyceride (MCT) oil may provide an immediately oxidisable “bypass” substrate to stabilise the bioenergetic crisis [60]. L-carnitine supplementation remains controversial: while potentially beneficial in CPT II deficiency, it may exacerbate toxic acylcarnitine accumulation in VLCAD and LCHAD and should only be used under specialist supervision [61]. Close monitoring for hypoglycaemia is essential, as FAOD patients are particularly vulnerable during catabolic stress [62].

Acute management of mitochondrial disease emphasises reduction in metabolic stress and maintenance of continuous energy supply, and avoidance of agents known to negatively affect mitochondrial function (e.g., valproate, statins, aminoglycosides, propofol). Correction of metabolic derangements (lactate, electrolytes, endocrine abnormalities) and organ-specific supportive care are essential [63].

In severe rhabdomyolysis complicated by AKI or refractory electrolyte imbalance, continuous renal replacement therapy or high-flux haemodialysis may be required to enhance myoglobin clearance and preserve renal function. Serial CK monitoring and supportive care remain central across all disorders.

### 2.6. Long-Term Management of Persistent HyperCKemia

Persistent hyperCKemia requires a structured, disorder-specific approach incorporating tailored exercise prescription, metabolic dietary therapy, and avoidance of myotoxic medications. GSD II (Pompe disease) represents a distinct entity owing to its lysosomal storage disorder pathophysiology. Enzyme replacement therapy (ERT; alglucosidase alfa) remains the cornerstone of treatment, supported by respiratory care and a high-protein diet to mitigate progressive muscle wasting [64,65]. Management of GSD III focuses on maintaining normoglycaemia through frequent high-protein, cornstarch-enriched meals [66]. GSD V management is primarily behavioural, with patients educated on the “second wind” phenomenon; ingestion of sucrose 5–10 min before exercise has been reported to improves exercise tolerance [67]. In GSD VII, high-carbohydrate intake before exercise is not recommended due to inhibition of fatty acid oxidation (“out-of-wind” phenomenon); ketogenic or high-protein diets may be beneficial [68,69,70]. GSD IX management is generally conservative, emphasising avoidance of prolonged fasting and anaerobic exertion, with high-protein diets often recommended [71] (Table 1).

Management of CPTII deficiency centres on restriction of long-chain triglycerides (LCTs), use of MCT oil—particularly pre-exercise—and strict avoidance of fasting and cold exposure [73]. VLCAD deficiency requires more stringent fat restriction, with MCTs providing up to 10–25% of total caloric intake and high-carbohydrate intake mandated during illness to suppress lipolysis [74]. LCHAD deficiency follows similar principles but necessitates careful supplementation with measured essential fatty acids to prevent deficiency while avoiding metabolite toxicity, given associated retinopathy and neuropathy [75] (Table 2).

Long-term management of mitochondrial disease is supportive. A therapeutic trial of a “mitochondrial cocktail”—commonly including thiamine, riboflavin, coenzyme Q10, and L-carnitine—is frequently employed given favourable safety profiles and potential biochemical benefit. Thiamine and riboflavin act as cofactors for mitochondrial enzymes, coenzyme Q10 functions as an electron carrier and antioxidant, and L-carnitine facilitates fatty acid transport. L-arginine may reduce stroke-like episodes in MELAS through nitric oxide–mediated vasodilation [77], while taurine shows emerging benefit, particularly in MT-TL1 mutations, by stabilising mitochondrial tRNA and mitigating oxidative stress [78] (Table 3).

Management of myoadenylate deaminase deficiency remains largely supportive, although some patients report symptomatic improvement with D-ribose supplementation [84].

## 3. Discussion

HyperCKemia in adults is a heterogeneous finding with important diagnostic and prognostic implications. Elevated CK levels reflect not only the intensity and type of physical activity—particularly eccentric or unaccustomed exercise—but also metabolic stressors that precipitate bioenergetic failure. In metabolic myopathies, acute decompensation may be triggered by sustained exertion, fasting, infection, surgery, anaesthesia, or other catabolic states. Exercise may unmask latent disease, underscoring the importance of a detailed activity history. Clinical phenotyping remains indispensable. The temporal relationship between exercise and symptom onset provides critical diagnostic clues, such as the “second wind” phenomenon in GSD V and delayed rhabdomyolysis in carnitine palmitoyltransferase II (CPT II) deficiency. These distinctions guide interpretation of biochemical findings and inform the selection of adjunctive investigations, including the NIFET and acylcarnitine profiling.

Contemporary diagnostic strategies integrate early genomic testing with targeted biochemical and functional studies, reserving muscle biopsy for unresolved cases, suspected acquired myopathies, or when histopathological findings are expected to influence management. Early genomic testing is particularly relevant in patients with persistent unexplained hyperCKemia, early disease onset, a positive family history, or features suggestive of an inherited myopathy, and is less informative when clinical findings indicate acquired, inflammatory, toxic, or endocrine causes. Access to genomic testing varies across healthcare systems and may influence the sequencing of investigations. Electromyography and muscle MRI are valuable adjuncts to confirm a myopathic pattern, exclude neuropathic processes, identify disease-specific patterns of muscle involvement, and guide further evaluation; however, when suspicion for inherited myopathy is high, they may not be required prior to genomic testing, while both remain important when diagnostic uncertainty persists or an acquired neuromuscular disorder is suspected. Overall, the diagnostic pathway should be individualised based on clinical context, pre-test probability, and resource availability [85,86].

Despite advances in diagnostic tools, delays remain common. For example, GSD V—often signalled by persistently elevated CK—is frequently diagnosed many years after symptom onset, with a reported median diagnostic delay of 29 years (range 0–68) [87]. In late-onset Pompe disease, the median delay has been estimated at approximately 5 years [88], while mitochondrial disorders show a median delay of 3.67 years and a mean delay of 4.79 years from initial clinical manifestation [89].

Management of GSDs is primarily directed toward maintaining euglycaemia and preventing catabolic stress. During acute metabolic crises such as rhabdomyolysis, priorities shift to rapid provision of alternative energy substrates and supportive care. Although a high-protein intake can be beneficial in chronic management, temporary moderation may be considered during acute episodes to limit nitrogen load in the setting of myoglobin-induced renal stress. Overall, management is highly subtype-specific and aims to mitigate the underlying energy deficit [13].

In GSD II, ERT is the cornerstone of treatment. Creatine monohydrate supplementation has also been explored, with outcomes strongly dependent on dosage [90,91]. Low-dose supplementation (approximately 60 mg/kg/day) may yield modest symptomatic and functional benefits, whereas high-dose regimens paradoxically exacerbate symptoms [92,93]. Vitamin B6 (pyridoxine), a cofactor for muscle glycogen phosphorylase has shown potential benefit in isolated reports [94].

GSD V and GSD VII are distinct muscle-predominant forms of GSD. Limited evidence suggests that some individuals with GSD V may benefit from a higher fat and lower carbohydrate intake, although responses vary [95]. In addition, pre-exercise sucrose ingestion has been shown to improve exercise tolerance and may reduce the risk of rhabdomyolysis in patients with GSD V [68,96]. A Japanese case report described marked clinical and biochemical improvement in a patient with GSD V treated with pyridoxine (60–90 mg/day) [97], although broader evidence remains limited. In contrast, emerging data suggest that some patients with GSD VII may benefit from a low-carbohydrate ketogenic diet, which may help bypass the glycolytic block [69,70].

An emerging adjunctive strategy in selected GSDs is anaplerotic therapy with triheptanoin, a synthetic seven-carbon triglyceride that generates acetyl-CoA and propionyl-CoA, replenishing tricarboxylic acid (TCA) cycle intermediates depleted by metabolic blockades [98]. However, randomised, double-blind crossover trials in GSD V and GSD VII showed no improvement in exercise performance, oxidative metabolism, or metabolic biomarkers, despite modest increases in resting tricarboxylic acid (TCA) cycle intermediates [99,100].

Management of CPT II, VLCAD, and LCHAD deficiencies, is centred on minimising long-chain fatty acid flux through defective β-oxidation pathways while preventing accumulation of cytotoxic acylcarnitine species. Dietary intervention is fundamental, with restriction of long-chain triglycerides and substitution with MCTs, which bypass the carnitine shuttle and enter mitochondrial oxidation independently [76,101,102,103]. In CPTII deficiency, MCT supplementation—particularly when administered pre-exercise—provides an alternative oxidative substrate, while strict avoidance of fasting and cold exposure is essential given their potent stimulation of lipolysis [104]. VLCAD and LCHAD deficiencies typically require more stringent fat restriction, with MCTs contributing up to 25% of total caloric intake and high carbohydrate feeding used to suppress endogenous fatty acid mobilisation [105,106]. Management of LCHAD is further complicated by progressive retinopathy and neuropathy, necessitating cautious supplementation with essential fatty acids to prevent deficiency without exacerbating metabolite toxicity [107,108].

In contrast to its investigational role in GSD s, triheptanoin is approved in the United States for long-chain fatty acid oxidation disorders, where it provides an alternative energy substrate by bypassing defective β-oxidation pathways [109,110].

Despite the paucity of high-quality randomised controlled trial data on mitochondrial disorders, expert consensus and observational studies support a therapeutic trial of coenzyme Q10 and other mitochondrial cofactors, given their favourable safety profile and occasional clinical benefit [63,80]. Creatine monohydrate supplementation has been proposed as a means of improving cellular bioenergetics in mitochondrial disease, with a 2022 narrative review suggesting potential benefit across a range of mitochondrial disorders [82]. The role of L-arginine remains controversial. While IV arginine may alleviate symptoms during acute MELAS episodes and long-term oral supplementation has been hypothesised to reduce stroke-like episodes through endothelial effects [77], mechanistic studies have yielded conflicting results [111], and a 2022 systematic review found no clear clinical benefit in either acute or prophylactic settings [112]. More recently, taurine has emerged as a promising therapeutic agent, shifting from its traditional role as an antioxidant to that of a “translational modifier.” High-dose taurine supplementation has been shown to restore translation of Complex I subunits, improve oxygen consumption, and reduce lactic acidosis [113]. An open-label phase III trial in Japan demonstrated a significant reduction in stroke-like episode recurrence in patients with MELAS receiving high-dose taurine (9–12 g/day) over 52 weeks, alongside increased taurine modification of mitochondrial tRNA^Leu(UUR)^ [114].

Although RYR1 mutations have classically been associated with malignant hyperthermia (MH) susceptibility, recent work by Hiraide et al. (2024) has expanded the phenotypic spectrum to include individuals presenting with exercise-induced myalgia and familial hyperCKemia [115]. The identified missense variant disrupts sarcoplasmic reticulum calcium homeostasis, creating a latent metabolic vulnerability that may be unmasked by physiological stress. These findings highlight the need to consider RYR1-related disorders beyond anaesthetic risk and support genomic evaluation in families with otherwise unexplained hyperCKemia [116].

In myoadenylate deaminase deficiency, oral D-ribose supplementation has been proposed to facilitate adenine nucleotide pool repletion. A single doses of 4 g administered at the beginning of exercise has significantly improved exercise tolerance, and total daily intakes up to 50–60 g reported as well tolerated, although clinical benefit remains inconsistent [84].

Drug-induced myopathy should be considered when elevated CK levels or muscle symptoms occur in patients taking medications known to affect muscle metabolism. Drug-induced myopathy typically presents with proximal muscle weakness and CK elevation without the immune-mediated features seen in inflammatory myopathies and lacks the systemic or malignant associations linked to macro-CK type 2. Ancillary tests such as autoantibody panels, EMG, or muscle biopsy may be required to exclude autoimmune necrotizing myopathy or other primary muscle disorders [3].

Endocrine disorders are a recognised cause of mild-to-moderate, often asymptomatic hyperCKemia, with hypothyroidism being the most common endocrine cause. Other endocrine contributors include hyperparathyroidism, acromegaly, and Cushing syndrome. In addition, metabolic disturbances such as hyponatremia, hypokalaemia, and hypophosphatemia. CK levels typically normalise after correction of the underlying endocrine or metabolic abnormality, making targeted evaluation important in otherwise unexplained CK elevation [2].

Immune-mediated necrotizing myopathy is a rare inflammatory myopathy characterised by prominent muscle fibre necrosis. It typically presents with progressive, symmetric proximal muscle weakness and markedly elevated serum CK levels, with few extra-muscular manifestations. Evaluation typically includes myositis-specific autoantibody assays, muscle MRI, and muscle biopsy to support the diagnosis [4].

In oligo- or asymptomatic adults with persistent hyperCKemia, an underlying neuromuscular disorder should be considered. Dystrophinopathies may present with isolated hyperCKemia, particularly in female carriers of Duchenne muscular dystrophy or Becker muscular dystrophy, and other inherited myopathies can present similarly [47]. Early genetic evaluation is therefore recommended to detect subclinical inherited muscle disease.

Familial idiopathic hyperCKemia is generally benign, sometimes autosomal dominant [117]. Transient post-exertional CK elevation reflects physiological muscle injury and typically resolves within days, whereas persistent elevation despite rest should prompt evaluation for occult neuromuscular disease [2]. The term “idiopathic hyperCKemia,” introduced by Rowland et al., has become less applicable with advances in molecular diagnostics, which now attribute many such cases to subtle genetic or metabolic defects [118].

Given the rarity of inherited metabolic myopathies, therapeutic recommendations are supported by heterogeneous levels of evidence, ranging from randomised controlled trials to expert consensus and mechanistic rationale. The strength of evidence varies substantially across interventions. ERT and selected disease-specific treatments are supported by randomised trials and longitudinal outcome data. Dietary and metabolic interventions are informed primarily by cohort studies, physiologic evidence, and clinical experience. Nutritional supplements and mitochondrial therapies are widely used based on biological and mechanistic rationale and favourable safety profiles but lack robust controlled evidence, whereas emerging metabolic and anaplerotic approaches remain investigational. Accordingly, treatment decisions should be interpreted within the context of differing levels of evidence certainty.

Collectively, these observations underscore the necessity for disorder-specific, mechanism-driven management strategies, as extrapolation of therapies across metabolic myopathies may worsen metabolic instability and muscle injury.

Prognosis in metabolic hyperCKemia is heterogeneous and depends on the underlying disorder, severity of muscle involvement, and timeliness of diagnosis, ranging from benign courses to significant morbidity due to recurrent rhabdomyolysis, renal failure, or progressive neuromuscular impairment.

## 4. Conclusions

Clinicians should maintain a high index of suspicion for metabolic myopathies in adults presenting with unexplained, persistent, or exercise-induced hyperCKemia, particularly when accompanied by suggestive symptoms or a relevant family history. A structured clinical assessment, informed use of biochemical investigations, and early integration of genetic testing are essential to establishing an accurate diagnosis. In adults, a genetics-first approach is now preferred over routine muscle biopsy due to its non-invasive nature and diagnostic yield. Continued advances in molecular diagnostics and targeted metabolic therapies offer significant promise for improving outcomes in this diverse and often under-recognised patient population.

## Figures and Tables

**Table 1:** Management recommendations for myopathic glycogen storage diseases.

GSD Type	Primary Dietary Strategy	Key Management Recommendations	Important Restrictions	Refs.
Type II (Pompe)	High-protein diet	ERT is essential; increased protein intake supports muscle maintenance.	Avoid excessive simple carbohydrates that may promote glycogen accumulation.	[65,72]
Type III (Cori)	High protein + cornstarch	Frequent meals with complex carbohydrates; uncooked cornstarch to maintain euglycaemia; protein ~25% of caloric intake.	Avoid prolonged fasting; limit simple sugars causing rapid glycaemic fluctuations.	[71]
Type V (McArdle)	Carbohydrate support	Ingest sucrose 5–10 min before exercise to improve tolerance (“second wind”).	Avoid high-intensity or anaerobic exertion.	[25,67]
Type VII (Tarui)	High protein/ketogenic	Emphasise protein and fat as energy substrates; ketogenic diet may improve tolerance.	Glucose or sucrose supplementation is ineffective and may worsen symptoms.	[68,70]
Type IX	Frequent feeding	Uncooked cornstarch/extended-release cornstarch, and high-protein diet.	Avoid prolonged fasting and intense anaerobic activity.	[28]

Abbreviations: ERT, enzyme replacement therapy; GSD, glycogen storage disease.

**Table 2:** Dietary management of long-chain fatty acid oxidation disorders.

Disorder	Primary Dietary Strategy	Role of MCT Oil	Key Restrictions/Monitoring	Refs.
CPT II deficiency	Low fat, high carbohydrate	Alternative fuel; may be used pre-exercise	Avoid prolonged fasting (>12 h) and cold exposure.	[33,73]
VLCAD deficiency	Very low long-chain fat	Provides 10–25% of total energy	Restrict long-chain triglycerides to essential needs.	[74,76]
LCHAD deficiency	Low fat, frequent carbohydrates	Essential alternative energy source	Strict fasting avoidance; monitor for retinopathy and hepatic involvement.	[60,75]

Abbreviations: CPT II, carnitine palmitoyltransferase II; VLCAD, very long-chain acyl-CoA dehydrogenase; LCHAD, long-chain 3-hydroxyacyl-CoA dehydrogenase; MCT, medium-chain triglyceride.

**Table 3:** Common adjunctive supplements in mitochondrial myopathy.

Supplement	Proposed Role/Mechanism	Typical Dose Range	Refs.
Coenzyme Q10 (ubiquinol)	Electron carrier (Complex I/II/III); antioxidant	50–600 mg/day (in divided doses)	[63,79]
Riboflavin (Vitamin B2)	Precursor of FAD/FMN; cofactor for Complexes I and II	50–400 mg/day	[63,80]
L-Carnitine	Facilitates mitochondrial fatty-acid transport	50–200 mg/kg/day (in divided doses)	[63,81]
Creatine monohydrate	Enhances phosphocreatine stores for ATP regeneration	3–6 g/day	[79,82]
Thiamine (Vitamin B1)	Cofactor for pyruvate dehydrogenase	100–1000 mg/day	[83]
Alpha-lipoic acid	Antioxidant; cofactor for PDH and α-KGDH	300–600 mg/day	[83]
L-Arginine	Nitric oxide precursor; used in MELAS stroke-like episodes	150–300 mg/kg/day (in divided doses)	[63,83]
Vitamins C and E	Antioxidant support	Vitamin C: 50–200 mg/day Vitamin E: 100–200 IU/day	[63,79]

Abbreviations: FAD, flavin adenine dinucleotide; FMN, flavin mononucleotide; PDH, pyruvate dehydrogenase; α-KGDH, alpha-ketoglutarate dehydrogenase.

## Data Availability

All data is contained within this manuscript.

## Abbreviations

AKI, acute kidney injury; ALT, alanine aminotransferase; AMPD, Adenosine monophosphate deaminase; AST, aminotransferase; CK, creatine kinase; CPT, carnitine palmitoyltransferase; CPT II, carnitine palmitoyltransferase II; D10, 10% dextrose solution; EMG, Electromyography; ERT, enzyme replacement therapy; FAOD, fatty acid oxidation disorder; GSD, glycogen storage disease; GSD II, glycogen storage disease type II (Pompe disease); GSD III, glycogen storage disease type III; GSD V, glycogen storage disease type V (McArdle disease); GSD VII, glycogen storage disease type VII (Tarui disease); IMDs, inherited metabolic diseases; IV, intravenous; LCHAD, long-chain 3-hydroxyacyl-CoA dehydrogenase; LCT, long-chain triglyceride; LOPD, late-onset Pompe disease; LSDs, Lysosomal storage diseases; MAD, myoadenylate deaminase; MCT, medium-chain triglyceride; MELAS, mitochondrial encephalomyopathy, lactic acidosis, and stroke-like episodes; NGS, next-generation sequencing; NIFET, non-ischemic forearm exercise test; PEO, progressive external ophthalmoplegia; PFKD, phosphofructokinase deficiency; RC, Respiratory Chain; RYR1, ryanodine receptor 1; TCA, tricarboxylic acid; VLCAD, very long-chain acyl-CoA dehydrogenase.
